# Everybody's Page

**Published:** 1890-04-26

**Authors:** 


### Everybody's Page.

There is an Oriental Proverb which says, "With time and patience the mulberry leaf becomes satin."

To give a new name to a dog is always a difficult matter, but Charles Reade the novelist (at least so the story goes), found a characteristic name for one of his dogs. He called it "Tonic," because he said it was a mixture of bark, steal, and whine.

An example of how fertility is returned to the earth, is found in pure snow, which yields on analysis a considerable amount of ammonia. On one occasion, snow collected from an elevated point in Shropshire, by Dr. Thursfield, was found to yield a quantity which, with a fall of snow equivalent to an inch of rain, would very nearly amount to one-third of a pound of ammonia per acre.

We feel that our sympathies are rather with the official in Annam who is mentioned by the *Temps*, who said that he was tired of administering a territory which contained as many tigers as inhabitants. When we learn, too, that the inhabitants were of that courageous nature that they raised the tiger to the level of a divinity, and were good enough to confer titles of nobility on him in order to propitiate him, and furthermore would never attack him unless by elaborate stratagem, deep pits, and so on, we feel still more strongly for that official; he probably felt that in a short space of time the four-footed nobles would administer him.

This is a marvellous age, full of scientific discovery, and we of the nineteenth century are very much impressed with the advances we have made, and the superior elevation of knowledge we stand on compared to departed centuries. So it is rather hard when Professor Anagnostakis, of Athens, comes and wishes to drag away some of our glory by saying that we are not to claim the microbe theory as a new discovery. It's not a bit new, it can be traced to Hippocrates' days; antiseptic treatment is not a conquest of modern science, certainly not, it is at least twenty-two centuries old, and was applied in ancient days quite as vigorously as ever it is now.

It is an interesting fact that in Columbia, where potatoes form the chief food of the people, and where potato disease runs riot very often, that the greater the altitude at which the vegetable is grown, the less is it liable to the disease, and at 9,000 feet above the sea, it grows in a perfectly healthy state.

Since 1884 great and successful efforts have been made to drain the marshes on the coast in Italy, about fifteen miles from Rome. The difficulties in carrying out the work have been enormously increased, owing to the place being so unhealthy, as to necessitate all work being suspended for several months. It is interesting to learn that the men who are doing the work are members of a large association near Ravenna, who are employed especially for this sort of work. A price is agreed upon by the contractor, and the men, who are subject to no master, control their own food, wine, dwelling, and hours of work, and withal, are peaceful and sober, and of robust constitution.

Mr. Goschen's statement on the consumption of alcoholic drinks during the past year must be a rude shock to those who saw in the decreased consumption of the past few years a sign of sure if only gradual national regeneration. It is not pleasant to have it so very clearly brought home to us that increased prosperity brings with it a rush on the part of all classes to the bottle, and an undesirable ambition to "toast the prosperity of the country." The French Society of Temperance, thanks to the generosity of Madame Lunier, is offering a prize for the best work on the hereditary results of alcoholism and drunkenness, and the means to arrest and lessen these consequences. No prize would be adequate for the man who could answer this latter question satisfactorily. What can we expect when the so-called educated classes set such a refined example and debase themselves by drinking, and what seems even more degrading by "nipping" all day long; are we to blame those who have never even been taught to keep their minds and bodies pure? It seems horribly evident that thrift and providence do not go concurrently with national prosperity.

